# Low-fatigue, adjustable pressure garments at 10, 20 and 30 mm Hg reduce scar thickness and improve pliability

**DOI:** 10.1371/journal.pone.0327691

**Published:** 2026-01-20

**Authors:** Danielle M. DeBruler, Molly E. Baumann, Britani N. Blackstone, Megan M. Malara, J. Kevin Bailey, Dorothy M. Supp, Heather M. Powell

**Affiliations:** 1 Department of Materials Science and Engineering, The Ohio State University, Columbus, Ohio, United States of America; 2 Department of Biomedical Engineering, The Ohio State University, Columbus, Ohio, United States of America; 3 Department of Surgery, Wake Forest University School of Medicine, Winston-Salem, North Carolina, United States of America; 4 Shriners Children’s Ohio, Dayton, Ohio, United States of America; 5 Department of Surgery, University of Cincinnati College of Medicine, Cincinnati, Ohio, United States of America; 6 Center for Stem Cell & Organoid Medicine (CuSTOM), Cincinnati Children’s Hospital Medical Center, Cincinnati, Ohio, United States of America; Faculty of Medical Sciences of Minas Gerais, BRAZIL

## Abstract

Scarring remains problematic post-burn injury even with pressure garment therapy, the current standard of care for scar suppression. Unfortunately, there is no consensus regarding the minimum pressure magnitude required for efficacy. Thus, to fill this knowledge gap, the relationship between the magnitude of applied pressure and scar outcomes was assessed using a porcine burn-excise-autograft model. Scars were treated with adjustable, low-fatigue pressure garments fit to 10, 20, or 30 mmHg; control scars were untreated (n = 16/group). Scar contraction, thickness, histological appearance, and biomechanics were assessed, and garment fatigue was measured. At all magnitudes of applied pressure, use of garments significantly reduced scar contraction and thickness, and improved scar pliability and elasticity, versus controls. Utilizing adjustable, low-fatigue garments, pressures of 10, 20, and 30 mmHg were reliably maintained throughout the study, demonstrating the efficacy of pressure garment therapy at pressures as low as 10 mmHg. While 30 mmHg pressure garments significantly reduced contraction compared to 10 or 20 mmHg, these garments required greater adjustment of the garment to reach the target pressure, which in practice could necessitate more frequent garment replacement. The results showed that although greatest improvement was observed at 30 mmHg, pressure as low as 10 mm Hg is effective at reducing post-burn scarring if that pressure is maintained throughout the garment’s use.

## Introduction

Pressure garment therapy (PGT) has been used to prevent and treat burn scars since the 1960s with conflicting reports regarding efficacy. Several clinical studies have shown improvement in scar thickness and appearance after PGT [1–3], while others have shown little benefit with no evidence of improved pliability [1,3,4]. Several recent evaluations have shown differences in anticipated and actual applied treatment parameters as well as risk of bias in many studies, emphasizing the need for further research before drawing definitive PGT conclusions [5,6]. Differences in observed outcomes following PGT may be due to variations in a patient’s predisposition for scarring, patient adherence to PGT, or the pressure garment treatment protocol [7].

Variables of PGT protocols include the magnitude of applied pressures [1], the duration of therapy post-injury [8,9], how quickly post-injury therapy is initiated [9–11], and the properties of the material used [12]. A recent survey indicated that the common target pressures range from 15 mmHg to over 25 mmHg, with most centers not measuring applied pressure [5]. It has been hypothesized that pressures greater than capillary pressure (~25 mmHg) and the associated reductions in blood flow [13,14] reduce scarring by decreasing collagen synthesis [14,15] and/or increasing collagen lysis [16]. Consequently, it is common clinical practice to utilize garments fabricated to achieve 20–30 mmHg pressure [1,3,4,17,18]. A prior study showed magnitude-dependent reductions in thickness and erythema in scars treated with garments that applied low (10–15 mmHg) and high (20–25 mmHg) pressures, with higher magnitudes leading to greater scar improvements [3]. A similar study comparing normal (20 mmHg) and low (12 mmHg) pressure found reduced scar thickness with normal pressure [1].

Though there is some evidence for greater efficacy with higher pressure garments, maintenance of high pressures can be challenging. Several studies evaluated applied pressure over one month of garment wear and found higher starting pressures led to greater decreases in pressure felt on the scar after one month, up to 35% loss [1,3], though others have shown up to 50% loss in pressure over one month of wear [19]. In another study, 40% of garments dropped below the desired 20 mmHg after one week of wear, with all garments dropping by week 3 [19]. Patients need to remove garments daily to wash both skin and garments. Donning and doffing garments can lead to stretching out of the fabric and loss of elasticity over time, decreasing pressure applied at the skin interface [20]. Due to these inconsistencies, as well as low rates of patient adherence to the prescribed therapy [21,22], the role of pressure magnitude in PGT efficacy is difficult to assess.

As higher pressures have been associated with increased risk of side effects such as pruritis, maceration, and skeletal deformations [6,8,18,23], it is desirable to use the lowest effective pressure magnitude. The goal of this study was to investigate the isolated effect of different pressure magnitudes on scar development using a porcine model. In contrast to clinical studies in burn patients, the porcine model decreases inherent variation by controlling burn location, burn depth, and patient compliance. Low-fatigue adjustable garments were utilized to reduce the effect of garment fatigue on outcomes [24]. Autografted, full-thickness burn wounds were created on red Duroc pigs and treated with either 10, 20, or 30 mmHg of pressure. Control wounds did not receive pressure. Scar characteristics were monitored over 3 months including scar area, depth, morphology, and biomechanics.

## Materials and methods

### Animal care and wounding

Eight full-thickness burn wounds were created on the dorsum of each of eight juvenile female red Duroc pigs (64 burns total) as described previously [24–26] using a protocol approved by The Ohio State University Institutional Animal Care and Use Committee (#2015A00000004R4). Anesthesia was initiated with Telazol (Zoetis, Florham Park, NJ) and maintained during the procedure with isoflurane. The dorsum was shaved and cleaned using two alternating 2% chlorohexidine and 70% isopropyl scrubs (Butler Schein, Columbus, OH). A custom burn device [27] was used to produce four 1x1 inch burns on each side of each pig by placing a metal stylus heated to 200°C on the skin for 40 seconds. Burn eschar was excised and wounds were covered with a split-thickness autograft (expanded to a ratio of 1.5:1) harvested from the dorsum using a Zimmer Air Dermatome (Zimmer, Warsaw, IN). Wounds were dressed with bolsters formed using Hydrasorb® surgical sponges (Carwild Inc, New London, CT) soaked in sterile saline and held in place with sterile spandex and skin staples (Henry Schein, Melville, NY). Vetrap^TM^ (3M Healthcare, St. Paul, MN) and Elastikon (Johnson & Johnson, New Brunswick, NJ) were used to secure fiberglass casts (3M Healthcare) over the dorsum to protect wounds. A NOVAPLUS fentanyl patch (Watson Pharmaceuticals, INC, Parsippany, NJ) was applied to the ear of each pig for 3 days for pain management. Dressings were removed one week post-grafting. For all biopsy collection procedures, anesthesia was initiated and maintained as above, with pain managed using an intramuscular injection of buprenorphine immediately after the biopsy collection, and as needed following collection. At week 12, pigs were euthanized. First, a deep plane of anesthesia was established as above. Subsequently, concentrated potassium chloride was delivered intravenously resulting in euthanasia, which was confirmed by the absence of cardiac and respiratory function.

### Compression application

Immediately after dressing removal at 7 days post-injury, PGT was initiated by applying pressure garments circumferentially around the dorsum. Custom pressure garments were constructed using Powernet fabric (Darlington Fabrics, Westerly, RI) in a wrap design affixed with Velcro, allowing garments to be individually adjusted to deliver the desired pressure on each graft [24–26] (S1 Fig). Each pig had two sets of garments available at each stage of the experiment to permit laundering without interrupting treatment. Grafts were assigned to receive 10, 20, or 30 mmHg PGT or no PGT controls (n = 16 per group) in a stratified manner to equal number of replicates at each site (cranial to caudal) to remove anatomic location as a confounding factor in the outcomes. Note that the circumferential nature of the pressure garments requires that the wounds that are opposite each other along the spine receive the same magnitude of pressure (S1 Fig). Polyurethane foam was used in concave areas of scars to ensure even pressure distribution. Pressures under each garment were measured and recorded using a Kikuhime pressure sensor (MediGROUP, Melbourne, Australia) each morning (n = 16 per group). The sensor was placed under the garment and directly over the wound to measure pressure at the wound site. Garments were then adjusted to meet the target pressure daily at each site. Every three days garments were removed and replaced with freshly laundered garments and every 21 days a garment was exchanged for a new garment to accommodate the increase in size of the pig as they grew. Average daily initial pressure and target pressure *±* standard deviation was plotted for 21 days of each garment’s use (n = 16 per group). Percent change in pressure after daily use was calculated over 21 days for each garment set by subtracting the pressure measurement collected each morning after a day of wear from the target pressure and multiplying by 100. The average percent change in pressure for that group was then calculated for each day (n = 21 per group) and the average over all the days reported as average daily percent decrease in pressure ± standard deviation.

### Scar area

Scar area was quantified at 1, 6 and 12 weeks post-grafting to measure scar contraction. Scar margins were traced onto transparent sheets; tracings were scanned with a ruler in the field of view and imported into ImageJ (https://imagej.nih.gov/ij/). Scar areas were measured and normalized to the area of that scar at 1 week to obtain percent changes in area (n = 16/group). Percent area ± standard deviation is reported. In this model, pig growth over time normally results in a ~ 190% increase in skin surface area over the 12-week study period [26]. Therefore, any scars that increase in area <190% at 12 weeks have contracted.

### Scar morphology and total scar thickness

Biopsies were taken from the scars at 1, 6, and 12 weeks after wounding. Biopsies were embedded in optimal cutting temperature compound (OCT; Fisher Healthcare, Houston, TX), frozen, and cryosectioned at 10 μm thickness (n = 16 per group). Four unique sections per block, separated by at least 250 µM, were stained with Masson’s Trichrome (Sigma-Aldrich, St. Louis, MO) and imaged to observe overall tissue morphology. For the week 12 samples, sections were scanned with a ruler in the field of view and imported into ImageJ. Three measurements per section were taken from the top of the stratum corneum to the interface between dermis and subcutaneous fat and averaged, and the average values of the four sections were used to calculate an average value for each scar. These average values were collated for each group (n = 16/group) and reported as average depth ± standard deviation. At 12 weeks, one biopsy from each wound was fixed in 10% formalin (Sigma-Aldrich), embedded in paraffin, sectioned, stained with Picrosirius Red (Electron Microscopy Sciences, Hatfield, PA) and imaged with a polarized light microscope (Zeiss Axioskop Widefield LM, Oberkochen, Germany) to visualize relative collagen fiber size and orientation.

### Scar biomechanics

Scar biomechanics were assessed 12 weeks after grafting using a Biomechanical Tissue Characterization (BTC-2000) device (SRI Technologies, Franklin, TN) as previously described [28,29]. The BTC-2000 applies a vacuum to the skin surface and uses a laser to measure skin displacement in response to applied force. Several *in vivo* mechanical properties were extracted using this device, including elasticity (mm), laxity (mm) and stiffness (mmHg/mm). Average biomechanical properties ± standard deviation are reported (n = 16/group).

### Vascularization analysis

Cryosections were immunostained with rabbit anti-von Willebrand Factor (VWF, Sigma-Aldrich) and Alexa Fluor 594-conjugated Donkey anti-Rabbit IgG secondary antibody (Thermo Fisher Scientific, Waltham, MA) and counterstained with 4’,6-diamidino-2-phenylindole (DAPI, Thermo Fisher). Samples were imaged with an Olympus FV3000 confocal microscope (Olympus, Center Valley, PA) then imported into ImageJ for quantification. To reduce any selection bias, four unique sections per block (separated by at least 250 µM) were stained and a 2x2 mm section of the upper dermis of each sample imaged with the number and size of blood vessels quantified in this area. Any region showing co-localized DAPI and VWF staining was counted. Average values per block/sample were quantified and average number of blood vessels/mm^2^, and average blood vessel size in mm^2^, ± standard deviation are reported for each group (n = 16/group).

### Garment material mechanics

To assess changes in garment mechanics following use, one set of garments per pressure group (10, 20 or 30 mmHg) that underwent 7 days of wear time was removed from the experiment for mechanical testing. Additionally, a new, unlaundered garment and a garment that had been laundered 3 times but not worn were used as controls. Strips of fabric (2x4 cm, n = 6/group) were cut from each of the garments and loaded into a mechanical tester (TestResources, Sharkopee, MN). The strips were strained at 0.5 mm/s until the applied force reached 3.5 N. The strain required to reach 3.5 N was recorded, as well as the force generated when the material was at 10% strain. Data were compared to fresh garment material stretched to 10% strain and 3.5 N force, as well as unworn garment material that was laundered 3 times. Average strain at 3.5 N and average force at 10% strain ± standard deviation are reported.

### Statistical analyses

Statistical analysis was performed using SigmaPlot v15 (Systat Software Inc., San Jose, CA). Samples size was based on power analysis using data from prior studies from our lab [24–26]. All studies had an n of 16 with the exception of the in vitro garment mechanics study where n = 6. Differences within each condition with time and among treatment conditions at the same time point were assessed using One-Way Analysis of Variance (ANOVA) with a *post hoc* test of Tukey for all data unless otherwise expressly stated. When datasets did not show equal variance as assessed by a Shapiro-Wilk test, as observed with all garment measurement studies, scar thickness data, area of blood vessels per field of view, elasticity and stiffness data, a One-Way ANOVA on Ranks with a Dunn’s *post hoc* analysis was performed. Statistical significance was established with a p value < 0.05.

## Results and discussion

### Applied pressure and garment fatigue

**Fig 1:**
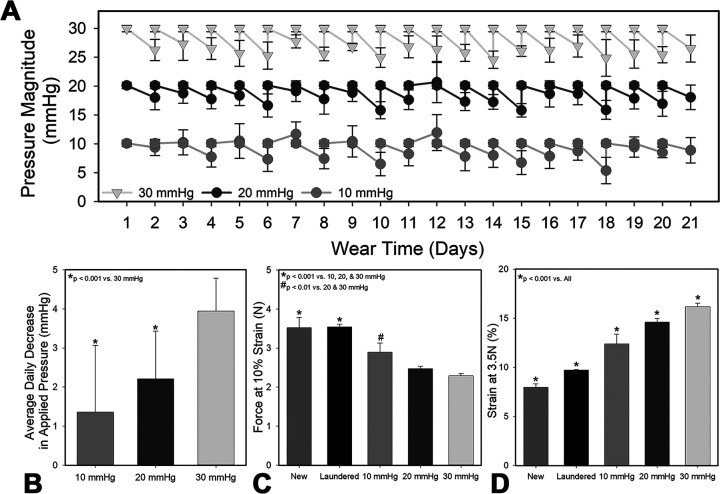
Garment fatigue is more pronounced with higher magnitudes of pressure. **A)** Pressure measured at the garment-skin interface immediately after garment application and after 24 hours of wear. Garments were adjusted to deliver the target magnitude each day. Measurements collected over a representative three-week period during the study showed no significant decrease in ability to reach target applied pressure and daily depreciations between 12-20% (n = 16). **B)** Average percent change in pressure over a 24-hour period. Garment fatigue was also assessed using tensile testing of strips of fabric obtained from new material and from garments that were worn for 7 days (n = 16). **C)** Garment fabric was strained to 10% and the resulting force measured (n = 6/group). **D)** Garment fabric was strained until a force of 3.5N was reached and the magnitude of strain required was recorded (n = 6). Garment fatigue was observed in all garments utilized at 10, 20 and 30 mmHg; however, fatigue was most pronounced when garments were designed to deliver 30 mmHg.

The magnitude of applied pressure below each garment decreased over 24 hours, requiring daily pressure adjustments. However, when measured pressure magnitudes were analyzed versus the total garment wear time, there were no observable decreases in pressure as garment aged (Fig 1A). When all 24 hour pressure drops were averaged over the course of 21 days, no significant difference in daily percent pressure reduction was observed (p > 0.05); garments worn at 10 mmHg dropped an average of 13.6 ± 17.1% over 24 hours, while 20 and 30 mmHg dropped 11.0 ± 6.1% and 13.1 ± 2.8%, respectively (Fig 1B).

The mechanical properties of used garment material were decreased compared to fresh material, and larger magnitudes of applied pressure were associated with increased degradation of mechanical properties (Fig 1C, 1D). New, laundered material required significantly more force to stretch to 10% compared to material obtained from garments employed at 20 and 30 mmHg (Fig 1C). Similarly, the strain required to reach 3.5 N of force was significantly less for new material than all other tested conditions (Fig 1D). While new material required a strain of 8.0 ± 0.34%, garments used to apply 10, 20 and 30 mmHg required 12.4 ± 1.0, 14.6 ± 0.37, and 16.1 ± 0.35% strain, respectively (Fig 1D).

The garments’ ability to undergo viscoelastic recovery is dependent on the fabric material, the magnitude and duration of pressure applied, and time allowed for fabric to recover before reapplying pressure [12,30,31]. In the current study, all garments were applied and allowed to recover for the same amount of time, thus differences observed in recovery can be attributed to differences in force applied. As the 30 mmHg group would require more strain to achieve the desired force, greater molecular rearrangement of the polymer chains and plastic deformation occurred, which likely could not be fully recovered and led to increasing strain needing to be applied to reach the target pressure. A prior study also observed a loss in garment recovery when higher loads were applied [31]. As a result, garments fit to apply 30 mmHg would likely require more frequent replacement or a change in the adjustable garment design to accommodate greater adjustment to reliably maintain 30 mmHg of pressure on scars.

### Scar appearance

**Fig 2:**
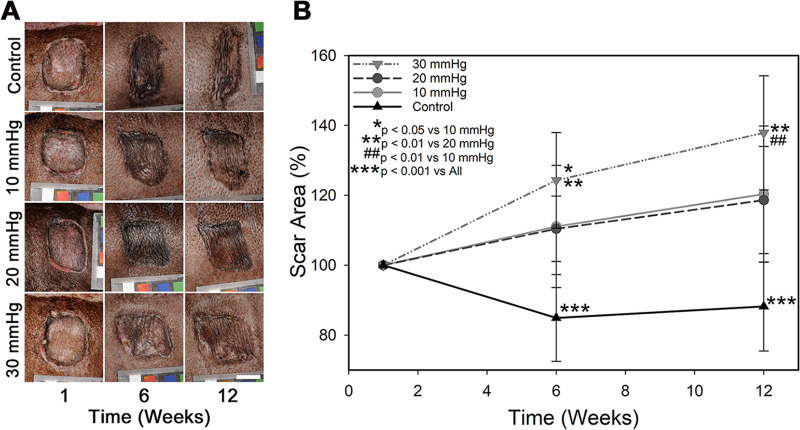
Scar appearance and contraction with time. **A)** Representative photographs of each treatment condition over time. Pressure treated scars were visibly smoother and less raised than control scars. Scale bar in the lower right is the same for all images (2 cm). **B)** Scar area as a function of time post-grafting. Area at each time point was normalized to the area of that individual scar at 1 week to obtain a percent change in area (n = 16). PGT at all magnitudes significantly increased scar area compared to controls (p, < 0.001), though 30 mmHg had significant improvements over 10 and 20 mmHg (p < 0.01).

Over time, control scars became rough, raised, and elongated with severe contraction in the cranial-caudal direction (Fig 2A). All pressure treated scars exhibited less contraction, maintaining a smoother appearance with a more equiaxial shape; however, scars receiving greater pressure magnitudes showed larger improvements. Scar area quantification at weeks 6 and 12 revealed that all pressure treated groups had significantly greater area than control scars (Fig 2B), indicating less scar contraction. The area of scars treated with 10 and 20 mmHg were similar to each other throughout the experiment, with average areas of 120 ± 19.5 and 118 ± 15.3% respectively at the final timepoint, compared to control scars that had an average area of 88 ± 12.8%. The area of scars treated with 30 mmHg was significantly greater than all other treatment conditions, with an average area of 137 ± 16.3% by week 12 (Fig 2B).

### Scar morphology

**Fig 3:**
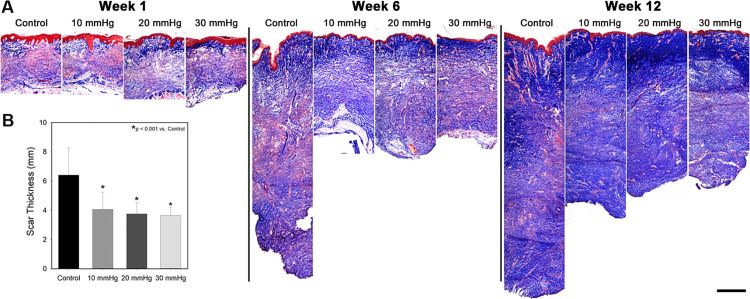
Scar anatomy as a function of time and treatment. **A)** Masson’s trichrome stained histological sections taken at 1, 6 and 12 weeks post grafting. Over time, collagen deposition increased in all groups (blue stain), though control scars tended to have a greater amount of collagen deposition in the deep dermis compared to pressure treated scars (scale bar = 1 mm). **B)** Scar thickness measured at 12 weeks post grafting (n = 16). All magnitudes of pressure significantly decreased the thickness of the scars compared to controls (p < 0.01).

**Fig 4:**
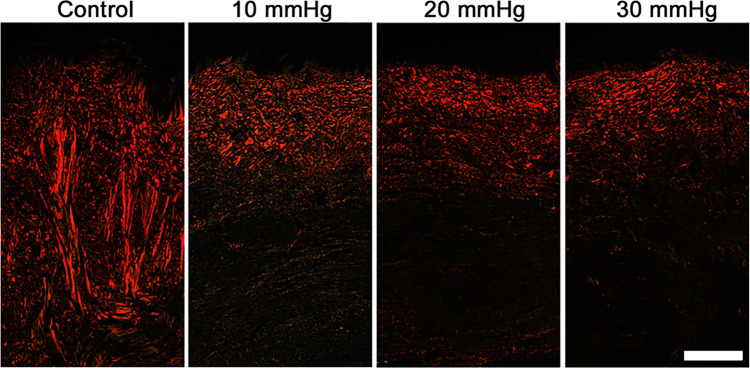
Representative Picrosirius stained histological sections taken 12 weeks post grafting. Collagen fibers in control scars tended to be thicker than pressure treated scars with a large proportion of fibers oriented perpendicularly to the surface of the scar (n = 16/group). Scale bar in the lower right is the same for all images (500 µm).

One week after grafting, trichrome stained histological sections showed minimal extracellular matrix (ECM) deposition (blue stain) with a large population of cells (red stain) in the dermis (Fig 3A). Mesh interstices were visible as areas with no ECM deposition in the upper dermis directly below the epidermis. Over time, ECM deposition increased in all groups, and evidence of mesh interstices was no longer visible by week 6. By 12 weeks post-grafting, control scars tended to have a greater amount of dense ECM deposition deeper in the dermis than pressure treated scars. Average total scar thickness in control scars was 6.4 ± 1.8 mm with pressure garments significantly reducing total thickness to an average of 3.6 ± 0.5, 3.8 ± 0.8, and 4.1 ± 1.2 mm in the 30, 20 and 10 mmHg groups, respectively (p < 0.001 vs. controls)(Fig 3B). No statistically significant differences were detected among pressure treated groups. Picrosirius red staining (Fig 4) revealed more collagen deposition in control scars compared to pressure treated scars. In addition, there was a large proportion of collagen fibers oriented perpendicular to the epidermis in control scars. In contrast, pressure treated scars showed substantially less collagen deposition that was concentrated in the upper dermis, with thin fibers that tended to be oriented parallel to the surface of the scars.

In the current study, scar thickness, pliability, elasticity, vascularity and contraction were all improved when PGT was utilized. Collagen organization within the dermis was less aligned and less dense when treated with PGT with no significant differences among magnitudes evaluated. PGT significantly reduced scar contraction over control scars, though 30 mmHg most significantly improved scar contraction over lower pressures. This may be due to differences in myofibroblast populations and contractility under different levels of pressure. Myofibroblast-like cell populations have been previously reported to undergo apoptosis when scars were exposed to physical pressure [32–35] and during realignment of collagen bundles [14,33]. Additionally, scar fibroblasts cultured under 20 or 40 mmHg were observed to undergo a reduction in number, TGF-β1 secretion, matrix deposition, and transdifferentiation to myofibroblasts versus cultures with no applied pressure [35]. When 40 mmHg was applied versus 20 mmHg, cell number and TGF-β1 secretion were more significantly reduced, suggesting larger magnitudes of applied pressure may lead to more drastic effects, especially in contractility [34].

### Scar biomechanics

**Fig 5:**
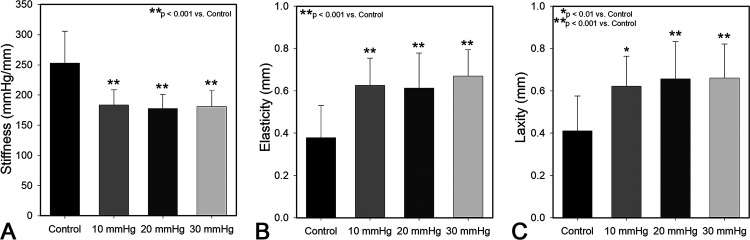
Pressure garment therapy at all magnitudes significantly improved scar pliability and elasticity. Biomechanical data obtained from BTC-2000 at 12 weeks post grafting. Control scars were significantly stiffer (p < 0.001) (A) with less elasticity (p < 0.001) (B) and laxity (p < 0.01 vs 10 mmHg, p < 0.001 vs. 20 & 30 mmHg)(C) than all pressure treated groups. Pressure treated groups were statistically equivalent (n = 16/group).

Pressure treated scars showed superior *in vivo* biomechanics compared to control scars (Fig 5). All three pressure magnitudes resulted in significant reductions in scar stiffness (Fig 5A) and increases in scar elasticity (Fig 5B) and pliability/laxity (Fig 5C) compared to controls. No significant differences among pressure treated groups were detected.

### Vascularization analysis

**Fig 6:**
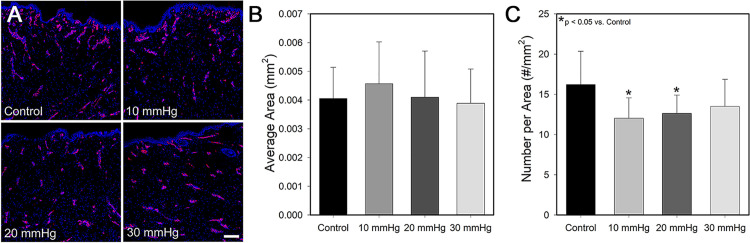
Blood vessel density is moderately reduced in scars treated with pressure garments at 10 and 20 mmHg. **A)** Representative histological sections of scars at week 12, showing blood vessels stained with VWF (red) and nuclei stained with DAPI (blue) (n = 16/group). Scale bar in the lower right is the same for all images (200 µm). Image analysis of blood vessels showed no difference in blood vessel size between groups **(B)**; however, there was a significant decrease in blood vessel number per area in the 10 and 20 mmHg groups compared to controls (p < 0.05) **(C)**.

No substantial difference in blood vessel size was observed between PGT treated scars and control scars; however, at week 12 scars treated with pressure garments appeared to have fewer blood vessels (Fig 6A). Upon quantification, there was no difference in the average size of blood vessels between treatment groups (Fig 6B). Scars treated with pressure magnitudes of 10 and 20 mmHg showed a small, but statistically significant decrease in the number of blood vessels per area present in the upper dermis compared to control scars (Fig 6C). Although scars receiving 30 mmHg of pressure tended to have less blood vessels than controls, this decrease was not significant likely due to the increased variability within that group.

Though it is hypothesized that pressures exceeding capillary pressure (~25 mmHg) are required to decrease blood flow to the area and collagen synthesis [13,15,16], the current data suggest that these magnitudes of pressure are not necessary for improved outcomes. Blood vessel density was reduced with 10 and 20 mmHg and all groups led to significantly thinner and less collagen-rich scars compared with untreated controls. It has been demonstrated that tension on a healing wound significantly increases scarring [36] and that mechanical off-loading can significantly reduce scar formation [37]. It is possible that the relationship between mechanical off-loading and scar reduction is not linear but plateaus at some level of strain reduction. This could lead to similar outcomes between the three groups tested in the current study. Additionally, the presence of tension within the developing scar may alter the susceptibility of collagen to degradation. Collagen degradation by both collagenase and matrix metalloproteinases has been shown to decrease under uniaxial tension [38,39]. The relationship between the magnitude of strain and change in degradation has been reported to be non-linear and varies depending on tissue type and composition [38,40–42]. Thus, the magnitude of strain reduction produced by 10, 20 and 30 mmHg may result in similar increases in susceptibility of the ECM to degradation.

## Conclusions

Low-fatigue, adjustable garments delivered a consistent amount of pressure to the scar over time and were used to evaluate the effect of different pressure levels on burn scars. Pressures ranging from 10–30 mmHg resulted in similar enhancements to scar pliability, thickness, vascularity and collagen formation. At all pressures utilized, scar contraction, thickness, pliability and elasticity were significantly improved over controls, with a significant improvement in contraction prevention observed in the 30 mmHg group compared to the 10 and 20 mmHg groups. The use of the adjustable garments was likely key to the efficacy observed at all pressures as the garments ensured that these pressures were consistent throughout the life of the garment. The integration of adjustable garments into routine clinical use is needed to improve effectiveness of pressure garment therapy.

## Supporting information

S1 Fig A) Schematic of wound sites and garment application. Pressure garments fit circumferentially around the torso of the pig treating one row of scars. All groups are present on each pig and row assignment among the cohort was stratified such that equal numbers of each group were assigned to R1-4. B) Photograph of the pressure garments on a pig. *Note:* The sites not covered by a pressure garment represent the control (0 mmHg) group. Loose garments, not exerting pressure on the tissue, could not be maintained on the body of the pig.
(TIF)
