# Supplementary figures and images for: Low-fatigue, adjustable pressure garments at 10, 20 and 30 mm Hg reduce scar thickness and improve pliability
Source: PLoS One. 2026 Jan 20;21(1):e0327691. doi: 10.1371/journal.pone.0327691 (PMC12818593; doi:10.1371/journal.pone.0327691)

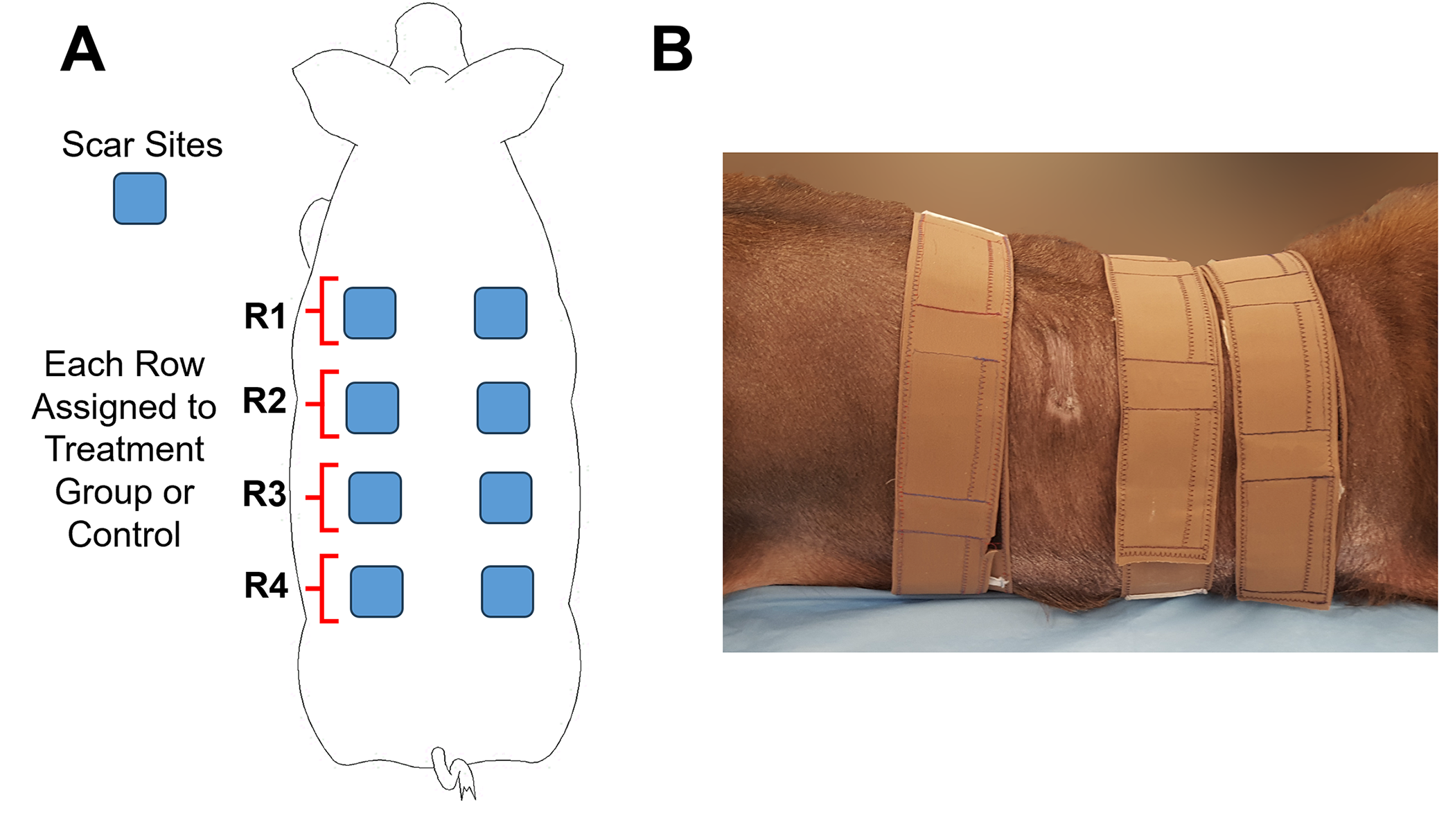

Supplement: S1 Fig — Pressure garments fit circumferentially around the torso of the pig treating one row of scars. All groups are present on each pig and row assignment among the cohort was stratified such that equal numbers of each group were assigned to R1-4. B) Photograph of the pressure garments on a pig. Note: The sites not covered by a pressure garment represent the control (0 mmHg) group. Loose garments, not exerting pressure on the tissue, could not be maintained on the body of the pig. (TIF) [file pone.0327691.s001.tif]
